# The ABCDs of ResUS – Resuscitation Ultrasound

**DOI:** 10.7759/cureus.4616

**Published:** 2019-05-07

**Authors:** Evan Avraham Alpert

**Affiliations:** 1 Emergency Medicine, Shaare Zedek Medical Center, Jerusalem, ISR

**Keywords:** cardiac arrest, hypotension, resuscitation, ultrasonography

## Abstract

Point-of-care ultrasound is ubiquitous in the hand of the clinician in many specialties, especially in the evaluation of the patient who is in shock or cardiac arrest. Two of the more common protocols are ACES (Abdominal and Cardiothoracic Evaluation with Sonography) and RUSH (Rapid Ultrasound for Shock and Hypotension). The purpose of this editorial is to introduce a new protocol that builds on many of the previous ones but is more comprehensive and easier to use. The ABCDs of ResUS - Resuscitation Ultrasound - is a systematic head to toe approach to facilitate the workup of the unstable patient. This includes viewing the A- Airway; B- Breathing (looking for pneumothorax); C- Cardiac, inferior vena cava (IVC), and the abdominal Core (Focussed Assessment with Sonography for Trauma- FAST exam); along with D- Deep vein thrombosis (DVT). Part or all of this protocol can be implemented based on the clinical circumstances to improve the diagnosis and management of critically ill patients.

## Editorial

Point-of-care ultrasound (POCUS) for the unstable patient is now ubiquitous in the hand of the clinician - whether they are an emergency physician, hospitalist, anesthesiologist, or intensivist. Many protocols have been developed to help the clinician evaluate and treat the patient who is in shock or cardiac arrest. One of the first was ACES (Abdominal and Cardiothoracic Evaluation with Sonography) [1]. This has essentially been replaced by the RUSH protocol (Rapid Ultrasound in SHock) [2]. Each of these focusses on using POCUS to rapidly evaluate and explain the reason for the patient’s clinical instability.

Recently there has been an attempt to evaluate POCUS in terms of its effect on patient outcomes. Atkinson et al. have made a robust effort to meaningfully evaluate the role of POCUS in cardiac arrest and hypotension. They have demonstrated the advantage of POCUS over electrocardiogram alone in the setting of non-shockable cardiac arrest [3]. In a multicenter trial, they have tried to answer the question as to whether POCUS can improve clinical outcomes in patients with undifferentiated shock [4]. The ultrasound protocol that was used in this study was the RUSH examination. This has been described conceptually based on the “pump,” “tank,” and “pipes.” The “pump” evaluates the heart looking for decreased ejection fraction, tamponade, or an enlarged right ventricle (concerning for pulmonary embolism). The “tank” evaluates the intravascular component of the patient including the inferior vena cava (IVC), abdomen (Focussed Assessment with Sonography for Trauma- FAST examination), and the chest looking for pneumothorax. The “pipes” view the arteries (including the aorta) as well as the femoral and popliteal veins for signs of deep vein thrombosis (DVT). For some, who find this conceptual approach difficult, there is a mnemonic: “HIMAP” (Heart, IVC, Morison’s, Aorta, and Pneumothorax).

However, the clinician, especially new learners, often get bogged down in this conceptual approach and sometimes miss performing the entire examination. The mnemonic formula is easier but finds the clinician moving the probe from the heart and abdomen, back to the lungs. There are attempts to introduce a more simplified standardized approach at least for the patient in cardiac arrest. The Cardiac Arrest Sonographic Assessment (CASA) exam is an example of this [5].

An even simpler protocol that is more comprehensive and easier to use is the “ABCDs of ResUS (Resuscitation UltraSound). This incorporates ultrasound for the patient in shock or cardiac arrest in a simple head to toe approach according to ABCD. “A” for “Airway” utilizes ultrasound in the intubated patient to verify that the endotracheal tube is in place. One looks for the single air-mucosa interface with comet-tail artifacts indicating tracheal intubation versus the double air-mucosa interface that signifies tube placement in the esophagus. The clinician then moves to point “B” (Breathing). The lungs are examined to determine whether there is a pneumothorax by seeing the absence of the lung sliding sign or the presence of the barcode sign (also known as the stratosphere sign), or if there are B lines which may indicate congestive heart failure. Then comes “C” (Cardiac) where the heart is examined for contractility, wall motion abnormality, tamponade, or right-sided enlargement. The IVC is evaluated at this point. One continues moving down the body to the abdomen. A FAST examination is performed and then the aorta is evaluated. This completes the ultrasound examination of the Core part of the body. The last point is “D” (DVT) where one can quickly examine the femoral and popliteal veins and to determine whether there is a thrombus that may be a cause of a pulmonary embolus.

Part or all of this protocol can be incorporated based on clinical circumstances. For example, the patient who arrives at the emergency department with hypotension and dyspnea who is subsequently intubated and continues to deteriorate may require the entire examination. A patient who is in pulseless electrical activity may just need the focused lung and heart component in order to evaluate the classic causes of cardiac arrest.
